# Characterization of the major autolysin (*AtlC*) of *Staphylococcus carnosus*

**DOI:** 10.1186/s12866-024-03231-6

**Published:** 2024-03-08

**Authors:** Maximilian Merz, Carolin J. Schiffer, Andreas Klingl, Matthias A. Ehrmann

**Affiliations:** 1 Chair of Microbiology, Technical University of Munich, Gregor-Mendel-Straße 4, 85354 Freising, Germany; 2 Plant Development, Department Biology I – Botany, Ludwig-Maximilians-Universität München, Großhaderner Str. 2-4, 82152 Planegg-Martinsried, Germany

**Keywords:** *Staphylococcus carnosus*, Autolysis, AtlC, Knockout mutant

## Abstract

**Background:**

Autolysis by cellular peptidoglycan hydrolases (PGH) is a well-known phenomenon in bacteria. During food fermentation, autolysis of starter cultures can exert an accelerating effect, as described in many studies on cheese ripening. In contrast, very little is known about autolysis of starter cultures used in other fermentations. *Staphylococcus (S.) carnosus* is often used in raw sausage fermentations, contributing to nitrate reduction and flavor formation. In this study, we analyzed the influence of PGHs of the strains *S. carnosus* TMW 2.146 and *S. carnosus* TMW 2.2525 on their autolytic behavior. The staphylococcal major autolysin (Atl), a bifunctional enzyme with an N-acetylmuramoyl-L-alanine amidase and a glucosaminidase as an active site, is assumed to be the enzyme by which autolysis is mainly mediated.

**Results:**

AtlC mutant strains showed impaired growth and almost no autolysis compared to their respective wild-type strains. Light microscopy and scanning electron microscopy showed that the mutants could no longer appropriately separate from each other during cell division, resulting in the formation of cell clusters. The surface of the mutants appeared rough with an irregular morphology compared to the smooth cell surfaces of the wild-types. Moreover, zymograms showed that eight lytic bands of *S. carnosus*, with a molecular mass between 140 and 35 kDa, are processed intermediates of AtlC. It was noticed that additional bands were found that had not been described in detail before and that the banding pattern changes over time. Some bands disappear entirely, while others become stronger or are newly formed. This suggests that AtlC is degraded into smaller fragments over time. A second knockout was generated for the gene encoding a N-acetylmuramoyl-L-alanine amidase domain-containing protein. Still, no phenotypic differences could be detected in this mutant compared to the wild-type, implying that the autolytic activity of *S. carnosus* is mediated by AtlC.

**Conclusions:**

In this study, two knockout mutants of *S. carnosus* were generated. The *atlC* mutant showed a significantly altered phenotype compared to the wild-type, revealing AtlC as a key factor in staphylococcal autolysis. Furthermore, we show that Atl is degraded into smaller fragments, which are still cell wall lytic active.

**Supplementary Information:**

The online version contains supplementary material available at 10.1186/s12866-024-03231-6.

## Background

*S. carnosus* is a coagulase negative staphylococcus and was first described by Schleifer and Fischer 1982 [1]. It is a starter culture for raw sausage fermentation, requiring nitrate reduction and improving flavor formation. Moreover, it is also proposed as a protein engineering platform [2].

Autolysis is defined as the self-digestion of bacterial cell wall. It is assumed that the lysis of a population or part of a population of cells is an evolutionary survival strategy of bacteria. The resulting release of nutrients, nucleic acids, and messengers serves survival, regulation of cell density, faster adaptability, and biofilm formation [3–6]. In lactic acid bacteria, also used as starter cultures in various fermented milk and meat production, autolytic systems have been described for several species. Among them are *Pediococcus* spp. [7], *Lactococcus lactis* [8], *Lactobacillus delbrueckii subsp. bulgaricus* [9] and *Latilactobacillus sakei* [10]. Data on the possible influence of bacterial autolysis on the quality of the fermentation product exist so far only for fermented dairy products. Thus, in the cheese model, a positive effect on flavor was described when selected starter cultures from *Lactococcus lactis* lyse during the ripening phase [11, 12].

Autolysins are a group of enzymes that degrade the peptidoglycan of the cell wall and are classified as peptidoglycan hydrolases [13]. Peptidoglycan hydrolases are a very large group of enzymes with a wide variety of functions. These enzymes include N-acetylmuramyl-L-alanine amidase, N-acetyl glucosaminidase, N-acetylmuramidase, and endopeptidase [14]. Amidases cleave the bond between the polysaccharide chain and the stem peptide [15]. The glucosidases cleave the polysaccharide chain [16, 17], and the peptidases cleave the crosslinking stem peptide between the polysaccharide chain [16].

The peptidoglycan of staphylococci consists of alternating N-acetylglucosamine and N-acetylmuramic acid. The carboxyl groups of N-acetylmuramic acid are linked to the stem peptide [18]. While the composition of the stem peptide is almost identical within staphylococci, there are slight differences in the interpeptide bridges. In *Staphylococcus aureus*, *Staphylococcus carnosus*, *Staphylococcus cohnii*, *Staphylococcus simulans*, and *Staphylococcus xylosus*, an L-Lys-Gly_5–6_ motif is found [1]. *Staphylococcus epidermidis* differs in the interpeptide bridges, where a significantly high amount of Glycin is replaced by L-Ser [19].

Peptidoglycan hydrolases are crucial for daughter cell separation, peptidoglycan turnover, control of peptidoglycan synthesis, and autolysis [17, 20]. *S. aureus* and *S. epidermidis* express different cell wall-associated and secreted autolysins. Among these, the most prominently described are the major autolysin AtlA from *S. aureus* and AtlE from *S. epidermidis* [21, 22]. AtlA and AtlE are bifunctional autolysins that are post-translationaly cleaved into two functional domains [21]: the N- acetylmuramoyl -L-alanine amidase (AM), which cleaves the amide bond between N-acetylmuramic acid and the stem peptide, and the endo-β-N-acetylglucosaminidase (GL) that cleaves the bond between N-acetyl-β-D-glucosamine and N-acetylmuramic acid [23]. Furthermore, the enzymes have repeat domains for localization and substrate recognition [24]. In addition to its role during cell division, AtlA in *S. aureus* also contributes to pathogenesis by interacting with multiple human extracellular proteins and mediating the release of bacterial extracellular DNA, which can promote biofilm formation and host colonization [25]. For *S. carnosus,* knowledge about the autolytic system and the major autolysin is limited.

In the current study, we investigated the autolytic system of *S. carnosus*, as it may influence the ripening and flavor of raw sausages. Therefore, we generated and characterized *atlC* mutants. For the generation of knockout mutants, we performed homologous recombination using the allelic exchange plasmid pIMAY* with counter-selection by pheS* [26, 27] . Although this system was initially developed for *S. aureus*, it has recently been demonstrated to function in *S. xylosus* [28]. However, this is the first time pIMAY* plasmid knockout mutants were generated in *S. carnosus*. By comparing mutant vs. wild-type strain behavior in different experiments, we demonstrated that the mutant’s autolytic potential is decreased, the cell morphology is different (cell daughter separation and peptidoglycan turnover), and the growth is reduced.

## Results

### Screening of the autolytic potential of different *S. carnosus* strains

**Fig. 1:**
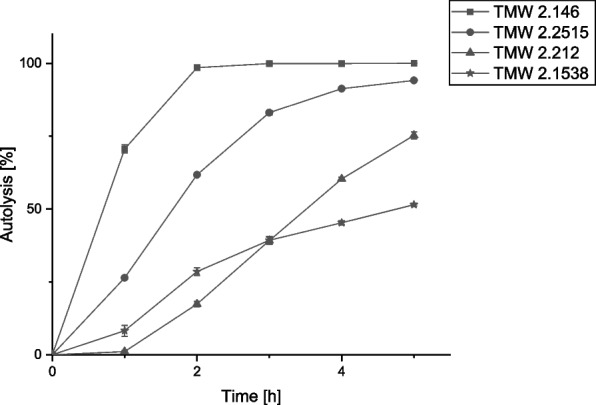
Screening of four *S. carnosus* wild-type strains for their autolytic potential. The autolysis of all four strains is measured over 5 hours including biological replicates. Autolysis is expressed as a percentage and was calculated from the OD_600_ values using Formula 1. The error bars show the standard deviation

In the first step, several strains of *S. carnosus* were tested for their autolytic potential. For this purpose, *S. carnosus* cells were transferred during the exponential phase to a buffer without nutrients [29]. Due to this stress factor and Triton X-100 as a trigger for autolysis [30], the cells start to lyse. This can be followed by a decrease in optical density over time. For better comparability, the OD_600_ values were converted into percentages using formula 1, which indicates the percentage of cells that have already lysed. These four strains were used because they originate from the raw sausage and genomic data are available. As can be seen in Fig. 1, the four strains all show different autolysis rates over the five-hour measurement. Strain TMW 2.146 is already completely lysed after 2 hours and thus indicates the highest autolysis of the tested strains. Strain TMW 2.2515 offers the second fastest autolysis, with 95% lysis after 5 hours. The other two strains tested showed lower autolysis after 5 hours, with 75% lysis (TMW 2.212) and 50% lysis (TMW 2.1538). For further work, strains TMW 2.146 and TMW 2.2515 were selected as they showed the highest autolysis.

### Bioinformatics analysis and selection of genes to be knocked out

**Fig. 2:**
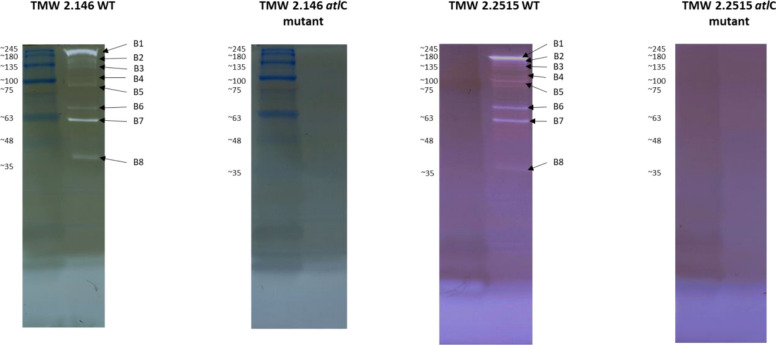
Zymogram of *S. carnosus* TMW 2.146 and TMW 2.2515 wild-type (WT) and the *atlC* mutant. Whole-cell hydrolysates are separated in zymograms according to their size, and then proteins are refolded in renaturation buffer (50 mM Tris -HCl pH 8 with 0.5% Triton X-100). The active lytic enzymes cleave the *Micrococcus lysodeikticus* cells in the gel, resulting in bright bands. The molecular sizes of the lytic bands can be determined by the size marker. In the WT, eight bands (B1 - B8) can be identified. No bands can be found in the knockout mutant

The genomes of *S. carnosus* TMW 2.146 and *S. carnosus* 2.2515 were assembled and annotated. Seven potential cell wall lytic proteins were identified by genomic analysis (Table 1). Seven potentially lytic proteins were selected based on the presence of cell wall lytic (glucosaminidase, amidase, muramoylhydrolase) or cell wall binding domains in their coding sequence. The calculated molecular masses of the seven proteins ranged from 30.08 kDa to 136.25 kDa. The predicted molecular weights were compared with those of bands obtained by zymography Fig. 2 of the wild-type Strains of TMW 2.146 and TMW 2.2515. For four of the potentially lytic proteins, the lytic bands in zymography matched the calculated protein mass, namely A2I62_09095 (*Bifunctional autolysin AtlC*) at 136.25 kDa, A2I62_12025 (*1,4-beta-N-acetylmuramoylhydrolase*) at 35.91 kDa, A2I62_00235 *(N-acetylmuramoyl-L-alanine amidase domain-containing protein*) at 80.07 kDa, and A2I62_09805 (*N-acetylmuramoyl-L-alanine amidase*) at 52.91 kDa.

**Table 1:** Seven potentially lytic enzymes were identified in *S. carnosus* TMW 2.146 by comparative genomics. Their locus tag, annotation, number of amino acids, and predicted molecular weight are given in the table. Four of the seven putative cell wall hydrolases were selected to perform a knockout. (+) knockout mutant available, (−) no knockout mutant available, (*) were not considered for the knockout

Locus tag (SAMN04563519)	Annotation	Number of amino acids	Molecular weight	Strain number	
				TMW 2.146	TMW 2.2515
A2I62_03315	LytD, Beta- N-acetylglucosaminidase	259	30.08 kDa	-*	-*
A2I62_05625	LytD, Beta- N-acetylglucosaminidase	281	32.22 kDa	-*	-*
A2I62_09095	Bifunctional autolysin Atl / N-acetylmuramoyl-L-alanine amidase (EC 3.5.1.28)/ endo-beta-N-acetylglucosaminidase (EC 3.2.1.96)	1254	136.25 kDa	+	+
A2I62_12025	1,4-beta-N-acetylmuramoylhydrolase	338	35.91 kDa	–	–
A2I62_00235	N-acetylmuramoyl-L-alanine amidase domain-containing protein	708	80.07 kDa	+	+
A2I62_06240	cell wall amidase LytH	291	32.7 kDa	-*	-*
A2I62_09805	N-acetylmuramoyl-L-alanine amidase	467	52.91 kDa	–	–

**Fig. 3:**
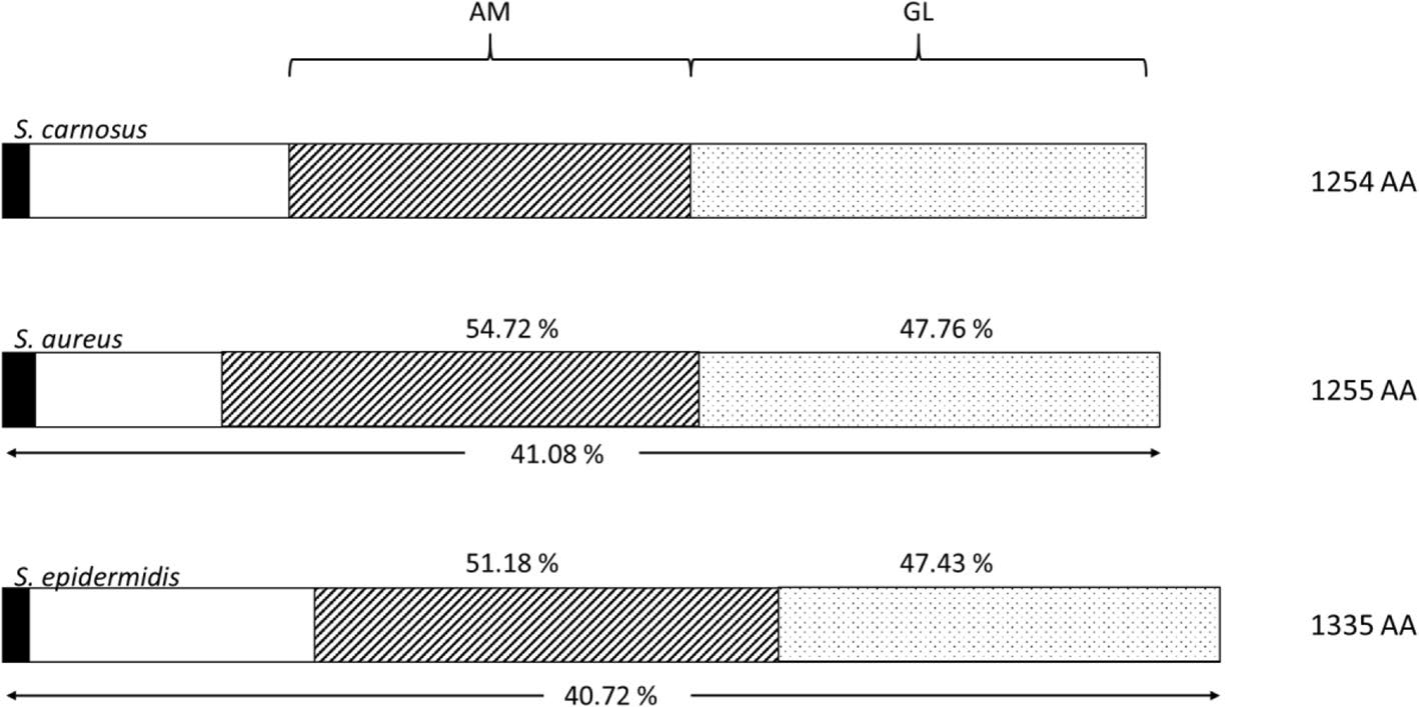
The Atl sequences of *S. carnosus* (TMW 2.146, TMW 2.2515) were compared with the sequences of *S. aureus* (NCTC 8325) and *S. epidermidis* (ATCC 14990) over the complete length and the two domains N-acetylmuramyl-L-alanine amidase (AM) and endo-β-N-acetylglucosaminidase (GL)

Furthermore, a protein sequence alignment was performed with the Atl’s of *S. aureus* NCTC 8325, *S. epidermidis* ATCC 14990, and the two *S. carnosus* strains TMW 2.146 and TMW 2.2515 to reveal differences between the different staphylococcus autolysins (Fig. 3). While Atl of the two *S. carnosus* strains are identical, they showed 41.08% similarity with Atl of *S. aureus* and 40.72% with Atl of *S. epidermidis*. The Atl of *S. aureus* shows 60.07% similarity with the Atl of *S. epidermidis*. This demonstrates a remarkable inter-species diversity. The two lytic domains N-acetylmuramoyl-L-alanine amidase (AM) and endo-β-N-acetylglucosaminidase (GL) were also compared. It was shown that the AM domain of *S. carnosus* matches 54.72% with *S. aureus* and 51.18% with *S. epidermidis*. In addition, the GL domain of *S. carnosus* matches with *S. aureus* with 47.76% and with *S. epidermidis* with 47.43%.

In a next step, an attempt was made to generate a knockout mutant for each of these four proteins in order to check which of the exhibited proteins is responsible for the observed autolytic activity. While knockout mutants of the bifunctional autolysin AtlC and the N-acetylmuramoyl-L-alanine amidase domain-containing protein could be generated, deletion of the genes encoding a 1,4-beta-N-acetylmuramoylhydrolase and a N-acetylmuramoyl-L-alanine amidase remained unsuccessful despite several attempts. Interestingly, no altered phenotype could be detected in the N-acetylmuramoyl-L-alanine amidase domain-containing protein mutant in subsequent experiments compared with the wild-type. Therefore, this mutant was not further addressed in this work.

### Growth monitoring of *Staphylococcus carnosus* WT and the *atl*C mutants

**Fig. 4:**
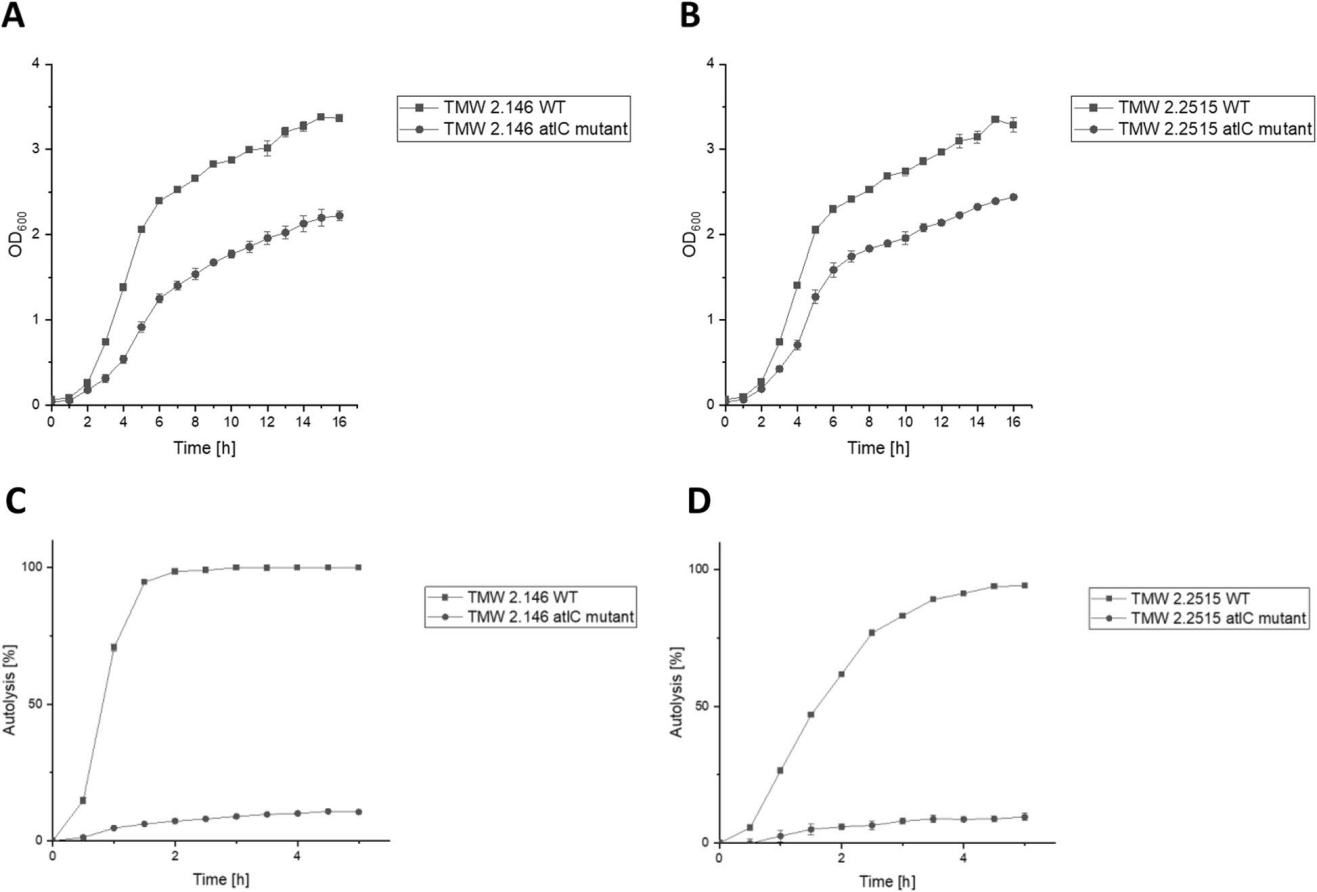
Growth curve and autolysis of *S. carnosus* TMW 2.146 and TMW 2.2515. Growth curve of *S. carnosus* strain TMW 2.146 (**A**) and *S. carnosus* strain TMW 2.2515 (**B**). Both wild-type strains are compared to their corresponding knockout mutant *atlC*. The OD_600_ was measured over 16 hours, and one measurement was taken every hour. The TSB media was inoculated to an OD_600_ of 0.05 and strains were grown at 37 °C with continuous shaking between the measuring steps. Autolysis of *S. carnosus* strain TMW 2.146 (**C**) and *S. carnosus* strain TMW 2.2515 (**D**). Both wild-type strains are compared with their corresponding *atlC* mutant. The autolysis of the strains is measured over 5 hours. Autolysis is expressed as a percentage and was calculated from the OD values using Formula 1

Knockout of the lytic enzyme AtlC may also result in altered growth of cells. For this reason, the growth of the wild-type and the *atlC* deletion strains was monitored over 16 hours, and the optical density was recorded every hour (Fig. 4 A and B). Both wild-type strains had similar growth behavior. After 5 hours, both reached an OD_600_ of 2 and after about 15 hours, they entered stationary growth. The growth curves of their mutant strains differed greatly from the growth curves of their respective wild-type strains. From the beginning, the growth is much slower than that of the wild-type. An OD_600_ of 2 is reached after about 13–14 hours. The final OD_600_ measured after 16 hours is also lower than the OD_600_ of the wild-type strains.

### Analysis of the autolytic potential of the *atlC* mutant and the wild-type

To show the influence of AtlC on the autolysis of *S. carnosus*, Triton X-100 autolysis assays were performed. In Fig. 4 C and D, the wild-type strain of *S. carnosus* TMW 2.146 and TMW 2.2515 is compared with its *atlC* mutant. In the wild-type strain of TMW 2.146, 95% of the cells lyse within 2 hours and then reach a plateau. In comparison, only 10% of the cells in the *atlC* mutant lyse within the first 2 hours, and 15% lyse after 5 hours of measurement. Likewise, in *S. carnosus* strain TMW 2.2515 (Fig. 4 D), the wild-type and the *atlC* mutant show a very similar behavior, yet there is a difference in the speed at which the cells autolyze. However, after 5 hours of measurement, the same autolysis values are obtained (95% of WT cells lysed vs. 15% of *atlC* mutant cells) as in strain TMW 2.146. These experiments clearly show that *atlC* influences the lytic behavior of *S. carnosus,* as the isogenic mutant strains exhibit a strongly reduced autolysis.

### Cell morphology of the WT and the *atlC* mutant

**Fig. 5:**
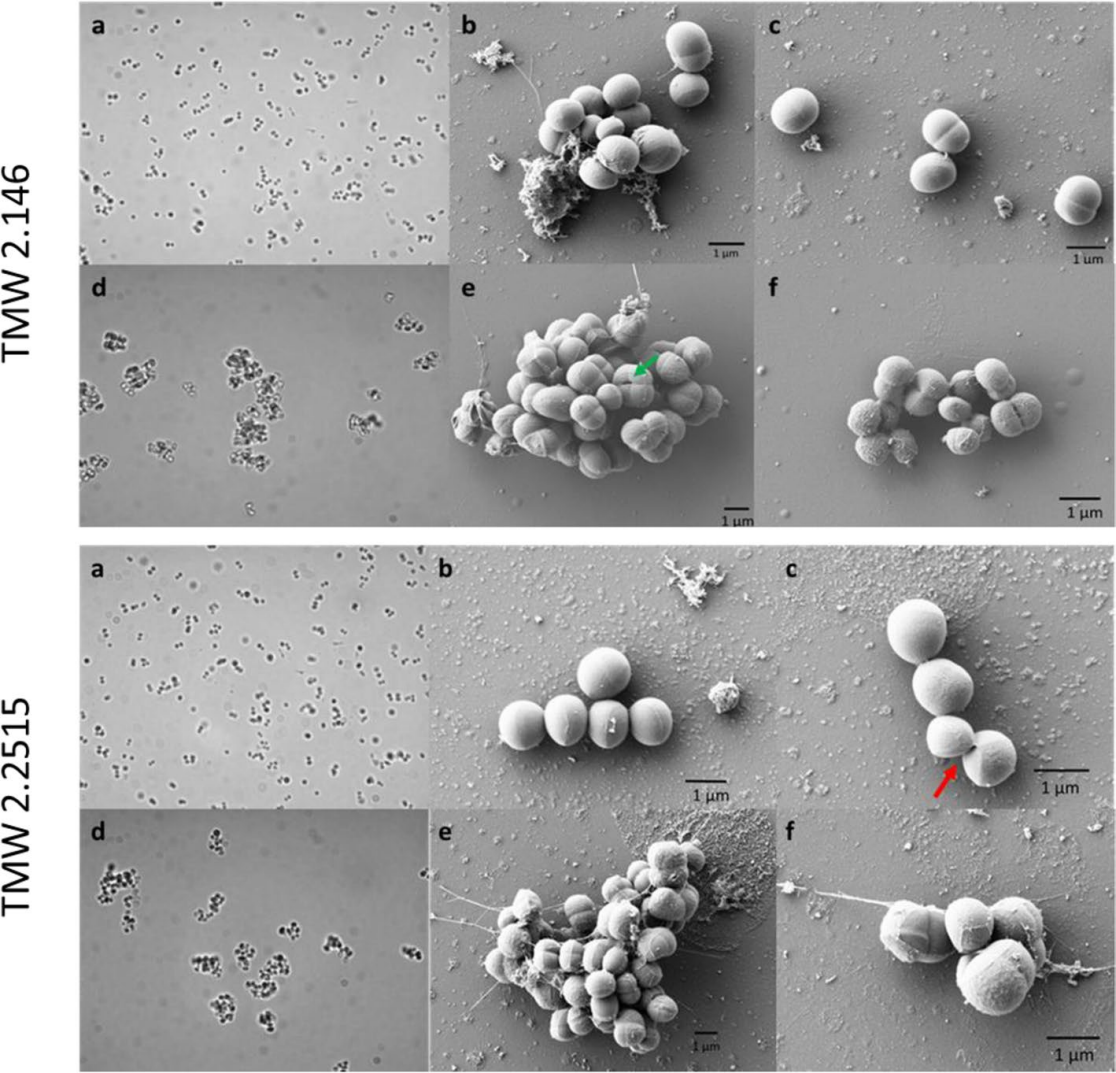
Light microscopy (a, d) and scanning electron microscope (b, c, e, f) of *S. carnosus* TMW 2.146 and TMW 2.2515 wild-type (a, b, c) and TMW 2.146 and TMW 2.2515 *atlC* mutant (d, e, f). The cells with the *atlC* knockout form larger cell aggregates than the wild-type, and the mutants also have a rough surface. The bar shows a length of 1 μm

To characterize changes in cell morphology, light microscopy and scanning electron microscopy (SEM) images were obtained of the *atlC* mutant and wild-type of *S. carnosus* TMW 2.146 and *S. carnosus* TMW 2.2515. Light microscopic imaging also observed a considerable difference between the wild-type and its respective mutants. While WT strains (Fig. 5 a) form smaller cell clusters typical for staphylococci with single cells also visible, mutant cells (Fig. 5 d) form very large cell clusters, and single cells are no longer visible. In the next step, scanning electron microscopic images were taken to further assess the structure of the cells. In the wild-type strains of TMW 2.146 and TMW 2.2515 (Fig. 5 b-c), smaller cell clusters and single cells can be seen in the SEM images. The surface of the cells is smooth and uniform. As indicated by the red arrow in Fig. 5 c, a dividing cell can be observed, with the daughter cell folding away from the parent cell. Very large cell clusters can be observed in the *atlC* mutants (Fig. 5 e-f) compared to the WT. Furthermore, the cells are not entirely separated from each other. The cell surface of the *atlC* mutants is rough and not uniform, indicating that *atlC* is also responsible for the regulation and turnover of the peptidoglycan. In addition, it can be seen that there is a second layer around some cells (Fig. 5 e, green arrow) holding the cells together. This layer is partially bursting, and single cells (green arrow) can be observed. In summary, imaging clearly showed that *atlC* is involved in daughter cell separation, as *S. carnosus* forms very large cell clusters when *atlC* is no longer present.

### Comparison of cell aggregation

**Fig. 6:**
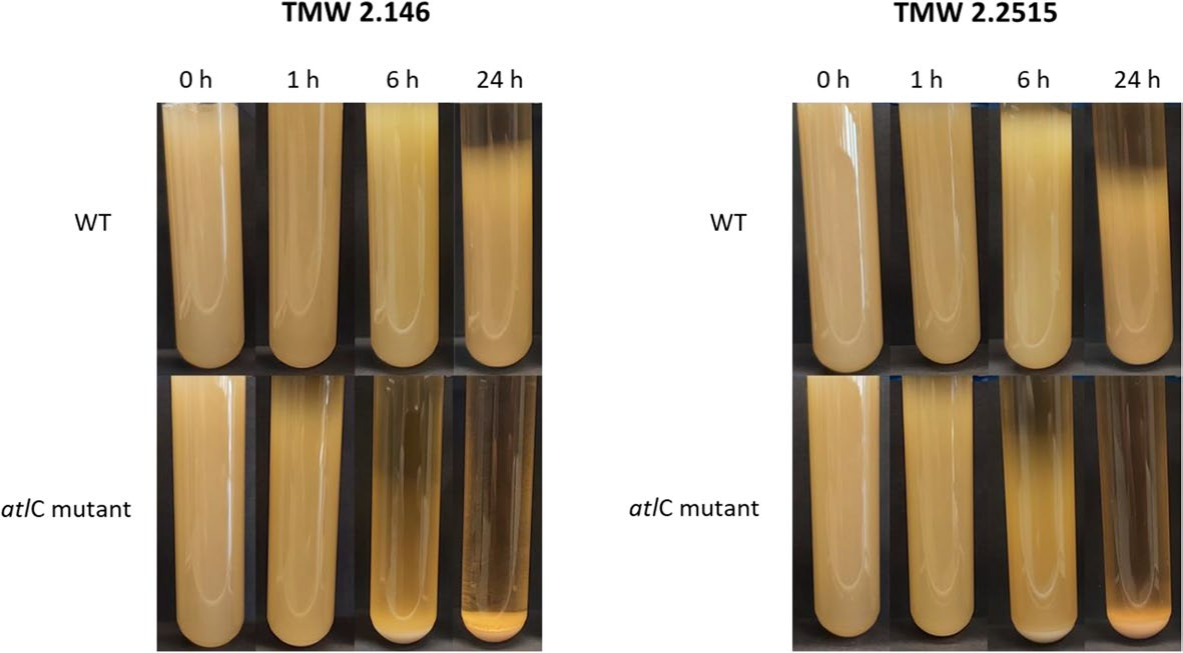
Sedimentation of *S. carnosus* TMW 2.146 wild-type (WT) and TMW 2.2515 wild-type (WT) was compared with the *atlC* mutant. For this purpose, cells were taken from an overnight culture, placed in cell culture tubes, and incubated statically. Over 24 hours, images of the cultures were taken at four time points (0 h, 1 h, 6 h, and 24 h) to document sedimentation. It can be observed that the cells of the knockout mutants of TMW 2.146 and TMW 2.2515 sediment faster than the WT cells of the same strain

Microscopic images showed that *atlC* mutants form very large cell clusters when they grow overnight in TSB medium. The preceding cell complex formation causes cells to settle faster in a liquid medium if not shaken further. To document this, a sedimentation assay was performed. Cells were grown under shaking conditions for 24 h. The cell suspension was then transferred to cell culture tubes, and cells were not shaken further. This allowed the cells to settle. Images of the cell culture tubes were taken after 0 hours, 1 hour, 6 hours, and 24 hours to record the sedimentation pace of the cells (Fig. 6). At the start time, the complete solution in the tubes is turbid. After the first hour, a clear separation area can already be seen in the upper part of the *atlC* mutants, while the entire solution of the WT is still turbid. After 6 hours, a quarter of the tube is already clear in the mutant, and after 24 hours, all cells have settled in the mutant. In the WT, only a quarter of the solution is clear after 24 hours of incubation. Here, a clear difference in sedimentation pace between the mutant and WT can be seen, likely due to the larger cell clusters formed by the *atlC* mutant.

### Zymography of WT and mutant strains

**Fig. 7:**
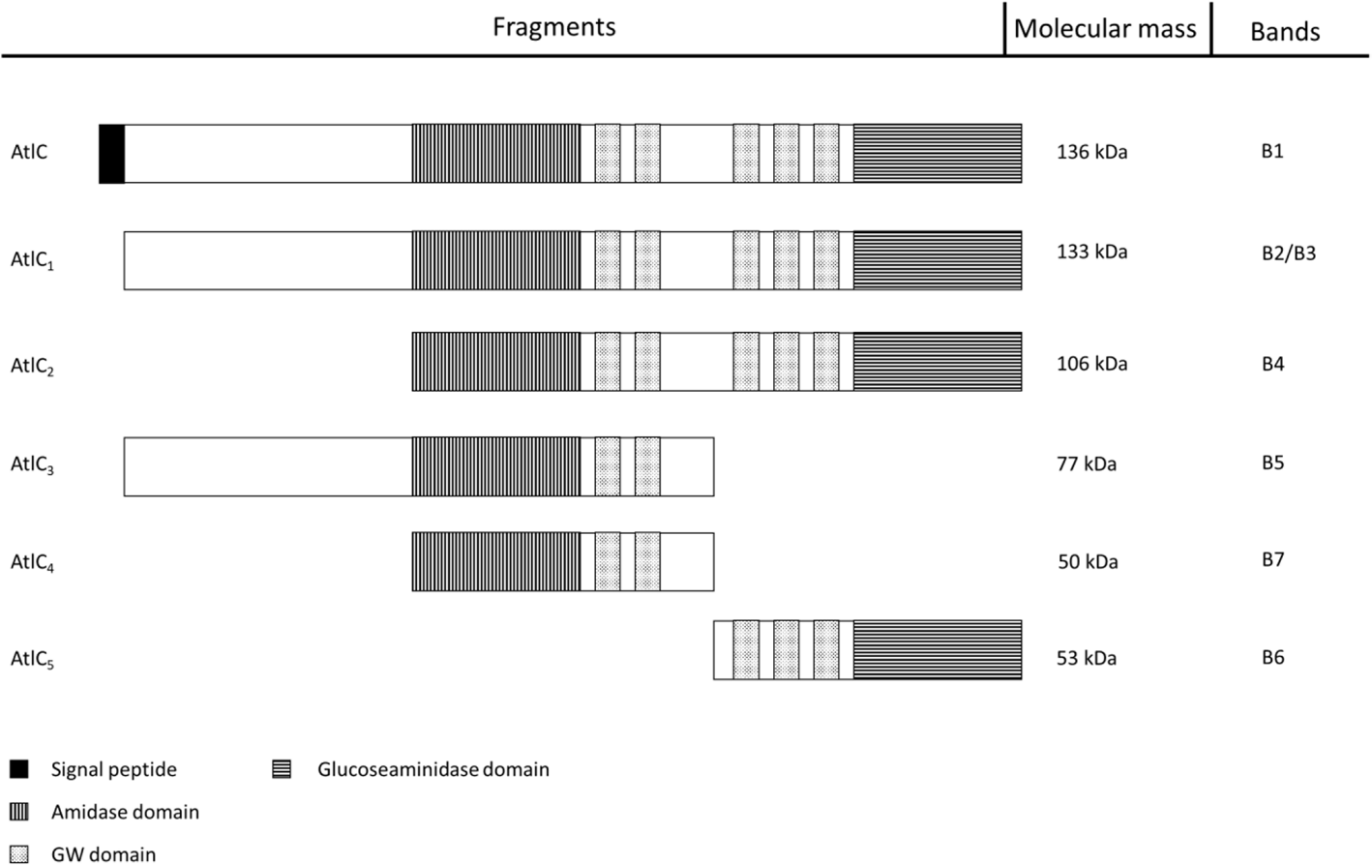
Atl is a bifunctional autolysin with different domains post-translationally modified. The complete protein (AtlC) with signal sequence has a length of 136 kDa. When the signal sequence is cleaved, the protein AtlC_1_ occurs with a length of 133 kDa. In the next step, the propeptide of the two active domains is cut off. AtlC_2_, with a size of 106 kDa, is formed. Then, the two functional domains (amidase and glucosaminidase) are divided, resulting in fragments AtlC_4_ and AtlC_5_ with a size of 50 and 53 kDa. In fragment AtlC_3_, the two active domains are separated first, leaving the amidase still attached to the propeptide. Fragment Atl_3_ has a size of 77 kDa. This is the hypothetical cleavage of the AtlC protein and the fragments that can arise and are active and thus can be detected in the zymogram. GW is a cell wall targeting signal and is named after a conserved Gly-Trp (GW) dipeptide

Zymograms are a common tool to detect cell wall lytic enzymes and to estimate their molecular size. For this purpose, whole cell extracts from overnight cultures of *S. carnosus* WT and *atlC* mutants were applied to zymograms. Cell wall lytic activity was indicated by hydrolyzing *M. lysodeikticus* cells, creating clear bands in an otherwise turbid gel. In this study, the gels were prepared with *M. lysodeikticus* cells to be able to find all lytic enzymes. A size marker was used to estimate the molecular weight of the hydrolytic enzymes. Eight bands are found in both WT *S. carnosus* strains (Fig. 2). The largest band had a molecular size of about 140 kDa, and the smallest band has a size of about 35 kDa. In between, another six bands were visible. No lytic bands were visible in the two *atlC* mutants, neither for TMW 2.146 nor TMW 2.2515. This suggests that all bands of the WT strain must originate from the major autolysin AtlC. Previous research on *S. aureus* and *S. epidermidis* already characterized the processing of the major autolysin (AtlA and AtlE) [31]. AtlC was theoretically split into smaller fragments in a further step, and the corresponding molecular masses were calculated (Fig. 7). The entire protein (AtlC), including its signal peptide, has a molecular size of 136 kDa. In the first step, the signal sequence was truncated (AtlC_1_), reducing the molecular size to 133 kDa. Next, the protein was further truncated by the propeptide (AtlC_2_), resulting in a size of 106 kDa. Finally, the two active domains are separated, resulting in an amidase domain (AtlC_4_) with a size of 50 kDa and a glucosaminidase domain (AtlC_5_) with 53 kDa. Before the propeptide is cleaved, the active domains can also be separated first, resulting in the fragment AtlC_3_ with a size of 77 kDa. This hypothetical band pattern was compared with the bands from zymography. This allowed some bands from the zymography to be assigned to the AtlC fragments. AtlC and AtlC_1_ fit to bands B1 and B3, respectively. Band B4 matches the fragment AtlC_2,_ and band B5 matches the fragment AtlC_3_. The two fragments AtlC_4_ and AtlC_5_ can be assigned most likely to bands B6 and B7. Furthermore, it is noticeable that additional bands are visible (B3 and B8) that cannot be assigned to any of the fragments. Band B8 is even lower than all other bands and smaller than the hypothetically determined bands.

### Change of the autolytic band pattern over time in *S. carnosus*

**Fig. 8:**
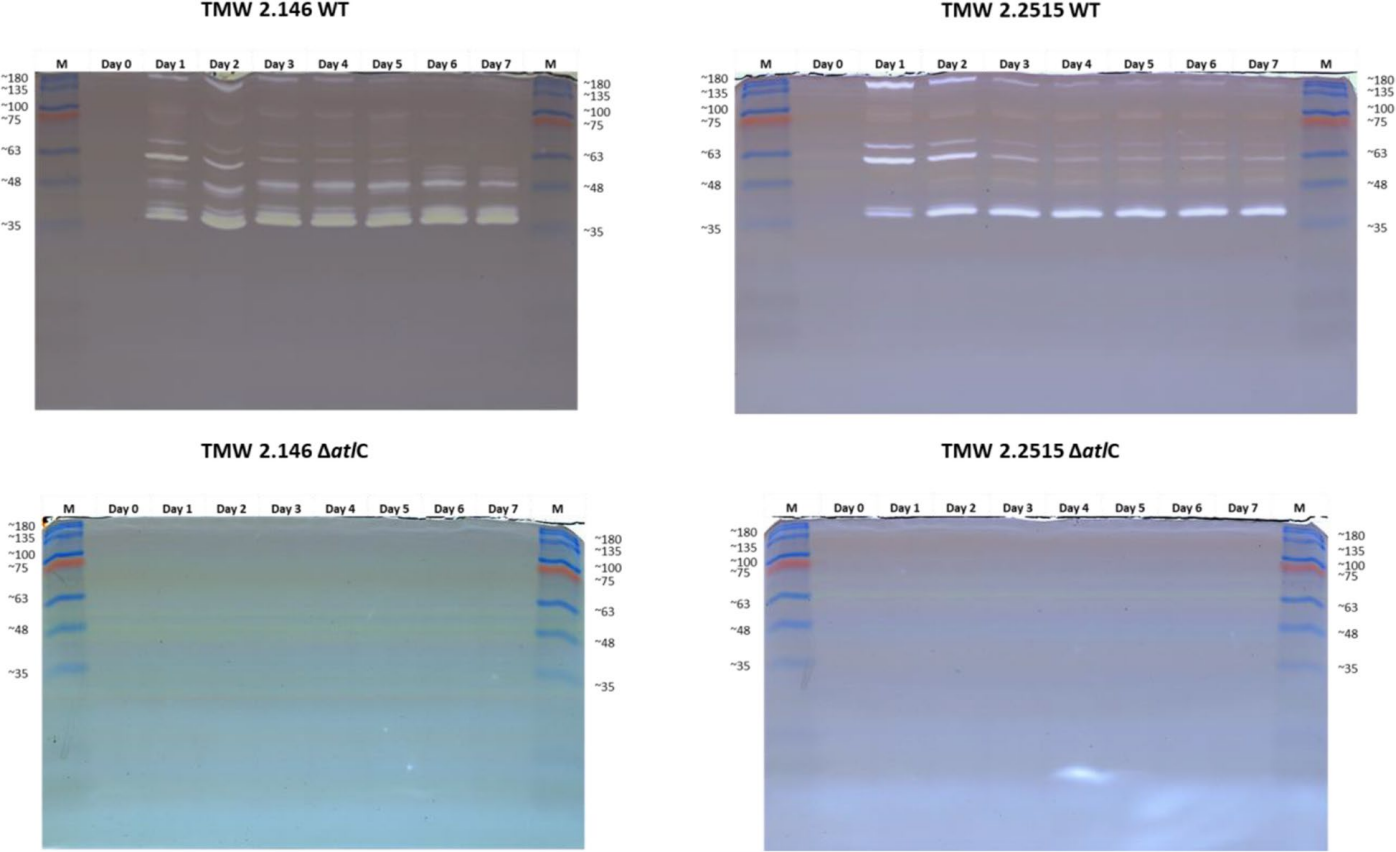
Zymograms of *S. carnosus* TMW 2.146 and TMW 2.2515 and their respective *atlC* mutants. Samples were taken from the supernatant of the respective cultures over 7 days. These samples were sterile-filtered and subsequently denatured by Laemmli loading dye. The denatured proteins were separated by size in a zymogram. Later, the proteins were renatured using a renaturation buffer (50 mM Tris -HCl pH 8 with 0.5% Triton X-100). Lytic proteins cleave *Micrococcus lysodeikticus* cells in the gel and form bright bands in the otherwise gray gel. First, many bands are formed in the wild-type strains, and no bands are visible in the knockout mutants. Furthermore, one can see that the banding pattern of the wild-type strains changes over the 7 days

To further refine the extent of extracellular protein involvement in autolysis, it was investigated whether any lytic activity is also contained in the supernatant of the bacterial culture. Therefore, zymograms were prepared from the supernatant of growing *S. carnosus* cultures (Fig. 8). As with the whole-cell protein extracts, the *atl* mutants of *S. carnosus* TMW 2.146 and TMW 2.2515 showed no lytic bands in the zymogram. However, a changing band pattern was seen over 7 days in the WT strains of TMW 2.146 and TMW 2.2515. The band pattern of the two strains also differs in intensity and number of bands. Comparing the banding pattern of strain TMW 2.146 (WT) from day 1 with the banding pattern from the whole cell protein extract, it is noticeable that bands B1 - B8 are found in both zymograms. However, additional bands are identified in the gel from the supernatant between 63 kDa and 35 kDa. Furthermore, the band pattern changes over the 7 days. Some bands become more intense, while others disappear entirely on day seven. In the gel of the TMW 2.2515 mutant a bright fleck can be recognized. This is not a band formed by a lytic enzyme but is an artifact of the SDS gel.

## Discussion

In this study, we describe the effect of an Atl deletion on the phenotype of *S. carnosus*. We focused on growth, cell morphology, and autolysis. Atl (AtlC) mutants were generated by homologous recombination using the allelic exchange plasmid pIMAY* [26, 27]. This system works very well in *S. carnosus*. Initially, four different genes were selected to be deleted, as all four could potentially impact the autolytic system of *S. carnosus*. However, in this study, it was not possible to transform the pIMAY* plasmid with the inserts for the 1,4-beta-N-acetylmuramoylhydrolase and the N-acetylmuramoyl-L-alanine amidase into *S. carnosus*. A possible reason could be that the transformed plasmid with the corresponding inserts was lethal for the cell, so only cells without the plasmid could be found. Reasons such as the transformation protocol, composition of the medium, or the competence of the *S. carnosus* TMW 2.146 cells can be excluded since it worked for two of the four plasmids.

Mutants of bifunctional autolysin Atl (AtlC) and N-acetylmuramoyl-L-alanine amidase domain-containing protein could be generated, which was also proven by PCR. However, the characterization of N-acetylmuramoyl-L-alanine amidase domain-containing protein mutant did not reveal any phenotypic differences compared with WT (Data not shown). Neither the growth curve, autolysis, nor the phenotype under the microscope differed from the WT strain.

The data clearly show that deletion of the AtlC leads to an apparent decrease in autolysis; only very low autolysis is detectable compared to the WT. The WT strain lysed to 95% within the five-hour measurement period. As already described for *S. aureus* [21], this suggests that AtlC is also the main autolysin in *S. carnosus*.

It was also shown that AtlC influences the growth of the cells. By measuring the optical density of the wild-type strains and the atl mutants, it could be shown that the growth of the atl mutants is slower than that of the wild-type strain. This can be explained by the fact that the enzyme is also used for daughter cell separation. If cell division is inhibited, the bacteria can no longer grow properly. However, it should be noted that the inhibition of cell division makes it difficult to determine growth by optical density. Since the cells form very large clusters (microscopic images), determining the optical density can lead to large errors. Takahashi et al. 2002 [32] found no significant differences in growth between the WT and the *atlA* mutant of *S. aureus*. In contrast, Bose et al. 2012 [25] found slight differences between the *atlA* mutant and the WT of *S. aureus*, which they also attributed to the difficulty of OD_600_ measurement. Therefore, the growth curves here are only intended to indicate how well the bacteria can grow and should not be taken as the absolute growth of the mutants.

The microscopic images show that the cells can no longer divide entirely from each other due to the knockout of *atlC*. The daughter cells remain attached to the mother cell. In *S. aureus*, AtlA localizes to the peripheral ring region, where the daughter cells detach from the mother cell [33]. However, it has also been shown that cells can divide despite not possessing AtlA. It is suggested that the division has mechanical reasons. Due to the high tension on the peripheral ring, the two cells burst apart [34]. Furthermore, the surfaces of the cells are rough. All this suggests that AtlC in *S. carnosus* is also required for daughter cell separation and peptidoglycan turnover. Due to the knockout, this no longer works, and the cells form large cell clusters. For AtlA (*S. aureus*) and AtlE (*S. epidermidis*) mutants, significant differences in cell division and cell morphology could also be shown. The mutant strains show large cell clusters, and the cell surface was rough and unstructured [22, 23, 35]. For lactic acid bacteria, it is known that due to the failure of one autolysin, its function is partially taken over by other lysins. This has been shown in *Lactiplantibacillus plantarum*, which has a main autolysin, and a second lysin is partly involved in cell division and autolysis [36]. This appears to be different from *S. carnosus*.

The sedimentation assay here again mirrors the results from the microscopic images. Due to the inhibited cell division, large cell clusters form, causing the cells to sediment faster than their wild-type counterparts, which form only small cell packets or single cells. Biswas et al. 2006 [23] also showed higher sedimentation for the AtlA mutant of *S. aureus* than the wild-type.

In the zymograms of the AtlC mutants, no bands could be found. This suggests that AtlC is the only enzyme involved in peptidoglycan lysis in *S. carnosus*. By comparing the results with the data published by Heilmann et al. 2005 [37] and Heilmann et al. 2003 [38], it is noticeable that *S. aureus* and *S. epidermidis* possess another autolysin. In the zymograms of the AtlE (*S. epidermidis*) and the AtlA (*S. aureus*) mutant, another band with 35 kDA was found in each case, which is not found in *S. carnosus*. This additional band is referred to as Aaa in *S. aureus* and Aae in *S. epidermidis* and is also involved in cell division [37, 38].

An interesting result can be seen in the zymograms from the bacterial culture medium over several days. The banding pattern is different from that obtained from the whole-cell protein lysates. In addition, the band pattern changes over the days, some of the bands becoming much stronger in intensity while other bands become weaker. Since the bands are not found in the *atlC* mutant, we assume they are derived from AtlC. However, here we come to the problem that the molecular masses of the newly formed bands do not match the postulated masses that arise when AtlC is processed. Therefore, we assume that AtlC is further cleaved, and these smaller fragments remain lytic active. AtlC is a bifunctional protein with two active lytic domains. It is cleaved post-translationally to become lytically active. This also produces the two active domains with an amidase and glucosaminidase function. We assume that these two peptides can be split into even smaller fragments in the further course and that these are further active to cleave the peptidoglycan. Gazzola und Cocconcelli 2008 [31] showed for *S. epidermidis* that the two active domains of AtlE can be degraded into two smaller fragments. It is known that two domains consist of active sites and still have two or three glycine-tryptophan domains (GWs). The GW domains serve to enable the enzyme to bind to the cell wall and then cleave it with the active site. It is known from *Latilactobacillus sakei* that its main autolysin has five binding domains; after cleavage of one of these binding domains, the enzyme remains lytically active [10]. Therefore, for the two domains of AtlC, we also assume that after the cleavage of one or two GW domains, the enzyme can continue to bind to the cell wall and thus continue to cleave it. Furthermore, it is not yet known how this cleavage takes place. Since we found all degradation fragments in the supernatant of the culture, we assume that these are also degraded extracellularly. With time all proteases of the cells can be found in the cell culture supernatant. One of these proteases could has the function to cleave the binding domains. Finally, the question arises whether the protein degradation is intended by the cell or occurs randomly since autolysin and protease meet through the lysis of the cell. GW domains are cleaved off during the degradation of AtlC. The GW domains are needed so that the AtlC can bind to the cell wall. It must therefore be assumed that the AtlC is no longer as lytically active as before with all GW domains. However, when 1 to 2 GW domains are cleaved off, the protein also becomes smaller and may therefore be able to bind better to the cell wall than before. This could also lead to an increase in lytic activity. However, these hypotheses would have to be substantiated by further experiments.

## Conclusion

Single deletion mutants of two potentially lytic proteins (A2I62_09095 and A2I62_00235) were made in *S. carnosus* TMW 2.146 and TMW 2.2515 in this study. The deletion of A2I62_00235 (N-acetylmuramoyl-L-alanine amidase domain-containing protein) did not result in an altered phenotype compared with the wild-type strain. We demonstrated that AtlC (A2I62_09095) is the major autolysin of *S. carnosus* and is significantly involved in autolysis and cell division. Without functional AtlC, daughter cells can no longer be separated, resulting in the formation of large cell clusters and likely impairing cell growth as a result. In addition, intensive processing of AtlC has been detected. These cleavage products are still active. Further work should characterize these fragments, how they arise, and whether the cleavage influences their lytic activity.

## Material and methods

### Bacterial strains and growth conditions

*S. carnosus* TMW 2.146, TMW 2.2515, TMW 2.212 and TMW 2.1538 were isolated from fermented raw sausages. All *S. carnosus* strains were cultivated in tryptic soy broth (TSB, soy peptone 15 g/L, casein peptone 15 g/L, and yeast extract 3 g/L, pH 7.2). For transformation experiments, *S. carnosus* was cultivated in brain heart infusion (BHI) and basic medium (BM, peptone 10 g/L, yeast extract 5 g/L, NaCl 5 g/L, glucose 1 g/L and K_2_HPO_4_ 1 g/L, pH 7.2). In all experiments, *S. carnosus* was aerobically cultivated at 37 °C and shaken at 200 rpm. *E. coli* DC10B is a DNA cytosine methyltransferase mutant of *E. coli* strain DH10B [26]. This strain was used for vector assembly, propagation, and purification. *E. coli* was cultivated in Lysogeny Broth ((LB), tryptone 10 g/L, yeast extract 5 g/L, NaCl 5 g/L) at 37 °C and 200 rpm shaking. If necessary, 20 μg/mL for *E. coli* and 10 μg/mL for *S. carnosus*, chloramphenicol (Carl Roth) was added to the medium.

### Triton X-100 induced cell autolysis assay

Autolysis assay of the *S. carnosus* strains was performed as described by Mani et al. 1993 [39]. 50 mL TSB medium was inoculated with 1% of an overnight culture of *S. carnosus*. The cells were collected when the culture reached the exponential phase (OD_600_ = 0.6–0.7) by centrifugation. The cells were washed once with ice-cold water and one time with 50 mM Tris-HCl (pH 7.2) and 0.05% (v/v) Triton X-100. Then, the cells were suspended in the same buffer to an OD_600_ of 0.6 to 0.8. The cells were incubated at 37 °C and 200 rpm shaking, and the OD_600_ was measured every 30 minutes. Autolysis is expressed as a percentage and was calculated using Formula 1. Here, OD_t = 0_ represents the OD_600_ value at the start time, and OD_t = x_ represents the measurement time points.

1 $$Autolysis\ \left[\%\right]=\left({OD}_{t=0}-{OD}_{t=x}\right)\ast \frac{100}{OD_{t=0}}$$

### DNA extraction, genome sequencing, assembly, and annotation

According to the manufacturer’s information, *S. carnosus* TMW 2.146 and TMW 2.2515 DNA were isolated with the E.Z.N.A. Bacterial DNA Kit (Omega Bio-Tek). The whole genome sequencing was performed with the Illumina HiSeq technology by Eurofins (Germany). Unicycler [40] and usegalaxy.eu was used to assemble the raw data. The NCBI Prokaryotic Genome Annotation Pipeline [41–43] was used to annotate the genomes. Genome sequences of *S. carnosus* TMW 2.146 and TMW 2.2515 were deposited at the NCBI database under accession numbers SAMN04563519 and SAMN36923051, respectively.

### Bioinformatics identification of autolytic enzymes in the genome of *Staphylococcus carnosus* TMW 2.146 and TMW 2.2515

Bioinformatics analysis, primer design, comparing protein sequences and comparative genomics were performed using CLC Main Workbench 8 software.

### Generation of the deletion mutants

For the deletion of the *atlC* gene (encoding the bifunctional autolysin) in *S. carnosus*, regions up and downstream of the to be deleted sequence (508 nt deletion) had to be amplified. Analogous to *atlA* in *S. aureus* and *atlE* in *S. epidermidis*, the *atl* of *S. carnosus* is referred to as *atlC* in this study. The Primers Atl1F (5‘-**ACTCACTATAGGGCGAATTGGAGCT**CAACAAATCAGTGTCCCTA-3‘), Atl2R (5‘- ATTTGATGGCTGCCAGAATCGCTGTATGTAAAC-3’), Atl3F (5‘- TACAGCGATTCTGGCAGCCATCAAATGTATC-3’) and Atl4R (5′-**GCTTGATATCGAATTCCTGCAG**CCATGCAAGATAGTCAGT-3′) were used for the amplification. The primer sequences were designed so that they fit both *S. carnosus* strains. The bold marked primer parts fit into the plasmid sequence (pIMAY*). The two PCR products were purified with the Monarch DNA Gel Extraction kit (New England Biolabs) from a 1% agarose gel. The vector pIMAY* [27] was digested with the two restriction enzymes SacI-HF and PstI-HF (New England Biolabs). The two inserts and the digested vector were assembled with the NEBuilder HiFi Assembly Master Mix (New England Biolabs) and then transformed by chemical transformation into DC10B *E. coli* competent cells (New England Biolabs). The successfully transformed cells were selected on LB plates with 10 μg/mL chloramphenicol. Plasmids were isolated with the Monarch plasmid DNA miniprep kit (NEB) and sequenced to verify the correct assembly of the vector. The right assembled vector was transformed by electroporation into competent *S. carnosus* TMW 2.146 and TMW 2.2515 cells [26]. Preparation of competent *S. carnosus* was done by the protocol Schiffer et al. 2021 [28]. *S. carnosus* TMW 2.146 and TMW 2.2515 was cultured overnight in BHI. On the next day, BM was inoculated 1% with the overnight culture and grew to an OD_600_ of 0.5 to 0.6. Afterward, the cells were harvested and washed twice with ice-cold water and another two times with 10% glycerol. Finally, the cells were suspended in 1/200 volume 10% glycerol with 500 mM sucrose and directly used for electroporation. For electroporation, 50 μl of the competent cells were mixed with 1 μg of plasmid and stored for 15 min on ice. Then, the mixture was transferred into a precooled electroporation cuvette (0.2 cm) and directly electroporated (2.5 kV). After electroporation, cells were directly mixed with 1 mL BHI with 200 mM sucrose and incubated for 1 hour at 28 °C. Afterward, the cells were plated on BHI plates with 10 μg/mL chloramphenicol and incubated for 2 days at 28 °C. Colonies were picked and checked with PCR (Atl1F and Atl4R) that the correct plasmid is inside. Colonies with the correct plasmid were used to perform the allelic exchange [27]. To prove the *atlC* knockout, PCR was performed using the primers Atl5F (5′-GTTTATCGATGGTGCTTG-3′) and Atl6R (5′-GCATAGTCGGCATAGTTA-3′). The primer binds on the genome of *S. carnosus* and amplifies the region which should be knocked out (Additional file 1).

### Growth characteristic

Growth curves of the wild-type and the mutant of the *S. carnosus* strains were monitored. Therefore, TSB was inoculated to an OD_600_ = 0.05 with an overnight culture. One mL of the cell suspension was transferred in a 48-well plate (Greiner) and incubated at 37 °C in a microplate reader (Sprectrostar^Nano^, BMG Labtech). Growth was measured by monitoring the OD_600_ every hour for 16 hours. Before each measurement, the plate was shaken for 5 min at 500 rpm. All measurements were done in technical replicates.

### Sedimentation assay

A sedimentation assay was performed to check the aggregation of the *S. carnosus* wild-type and the *atlC* mutants. The cell wild-type and *atlC* strains were grown in TSB for 24 h at 37 °C and 200 rpm. After 24 h, 5 mL of the cell suspension was transferred into a culture tube without shaking. Pictures of the wild-type and the *atlC* mutant were taken at four-time points (t_0_, t_1_, t_6_, and t_24_) to record the sedimentation.

### Screening for lytic enzymes in the supernatant

For the screening of lytic enzymes in the supernatant, 50 mL BM media was inoculated with 500 μl of *S. carnosus* TMW 2.146 or TMW 2.2515 or their respective mutants. Over 7 days with constant shaking at 200 rpm and 37 °C, samples were taken from the cultures. The samples were centrifuged for 10 min with 6000×g and the supernatant was filtered with a 0.2 μm filter. The filtrate was subsequently stored at − 20 °C until it was used for the zymography.

### Preparation of whole-cell protein extracts

For whole-cell protein extracts, 15 mL overnight bacteria cultures were used. The culture was centrifuged (6000×g, 10 min) and then suspended in 1 mL lysis buffer (10 mM EDTA, 10 mM NaCl, 2% (w/v) SDS, and 10 mM Tris-HCl pH 8.0). The bacteria suspension was boiled for 10 min at 98 °C and then centrifuged again. The Protein suspension can now used for the SDS-Page and the zymography.

### Zymography of whole-cell protein extracts and medium supernatants of growing cells

The lytic activity was determined on a 12% polyacrylamide-sodium dodecyl sulfate (SDS) gel containing 0.2% (w/v) *Micrococcus (M.) lysodeikticus* ATCC No. 4698 (Sigma Aldrich) [39]. The whole-cell protein extract and the supernatant were mixed 1:1 with Laemmli loading dye (Sigma Aldrich) and boiled for 10 minutes. After electrophoresis, the gel was incubated in 50 mM Tris-HCl pH 8 with 0.5% Triton X-100 for 24 hours at 37 °C.

### Light microscopy

Overnight cultures of wild-type and mutant strains were used to visualize cell morphology in microscopic images. A Zeiss Axiostar plus Microscope with an Axiocam ICc1 Zeiss camera was used.

### Scanning electron microscopy

Fixation for SEM was carried out as described previously [44]. Cells were prefixed with 2.5% glutaraldehyde in 75 mM cacodylate buffer also including 2 mM MgCl_2_ (pH 7.0). Afterward, the cells were applied to glass slides, covered with a cover slip, and rapidly frozen in liquid nitrogen. This was followed by detaching the coverslip and storing the glass slide in a fixation buffer overnight. The next steps were: four times washing with buffer (10, 20, 30, 50 min), post-fixation with 1% osmium tetroxide for 30 min, washing with buffer (20 min) and double distilled water (5, 15, 90 min). The samples were dehydrated in a graded acetone series, critical-point-dried, mounted on stubs, and sputter-coated with platinum. For imaging, a Zeiss Auriga crossbeam workstation (Zeiss, Oberkochen, Germany) with an acceleration voltage of 1.5 kV and a working distance of 5 mm was used.

### Supplementary Information

**Supplementary Material 1.**

## Data Availability

The genomes of TMW 2.146 and TMW 2.2515 are accessible under the accession number SAMN04563519 and SAMN36923051.
