# Supplementary material for: Characterization of the major autolysin (AtlC) of Staphylococcus carnosus
Source: BMC Microbiol. 2024 Mar 8;24:77. doi: 10.1186/s12866-024-03231-6 (PMC10921637; doi:10.1186/s12866-024-03231-6)
Supplement: Supplementary file 1 — Supplementary Material 1. [file 12866_2024_3231_MOESM1_ESM.pdf]

uncropped Gels Figure 7

TMW 2.146 WT

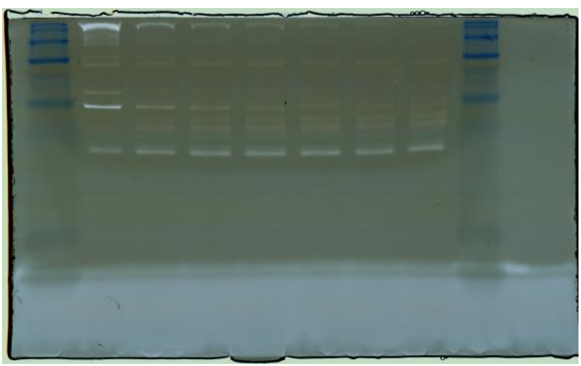

TMW 2.146 ΔatlC

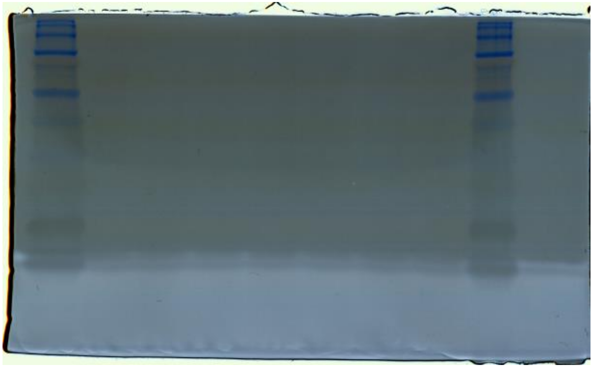

TMW 2.2515 WT

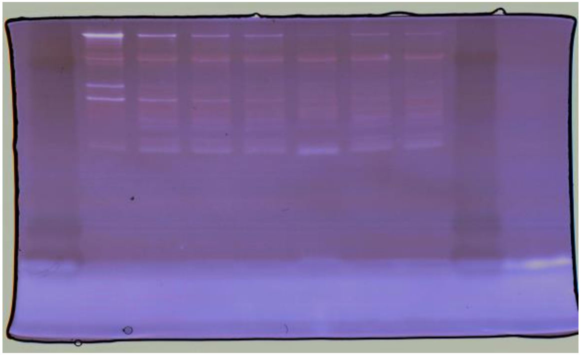

TMW 2.2515 ΔAtl

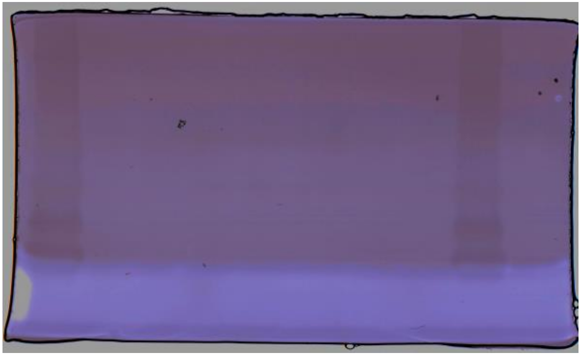

uncropped Gels Figure 9

TMW 2.146 WT

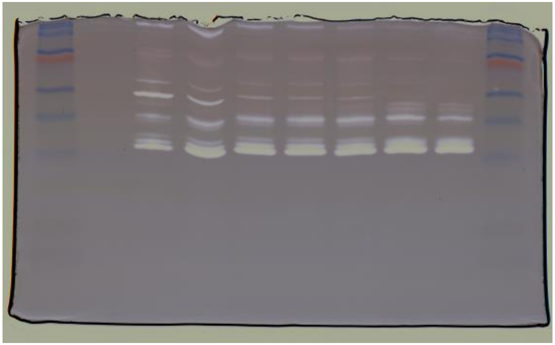

TMW 2.2515 WT

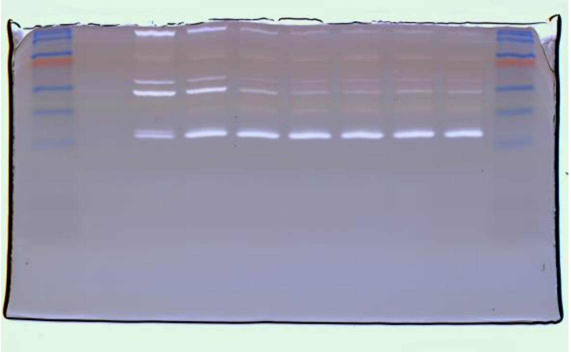

TMW 2.146 Δ*atl*C

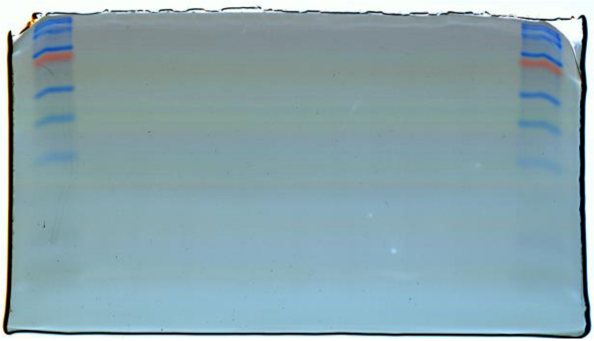

TMW 2.2515 Δ*atl*C

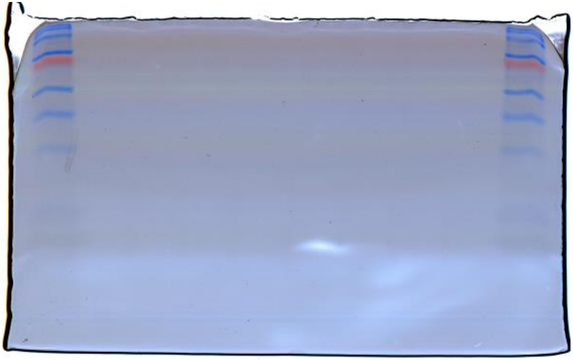

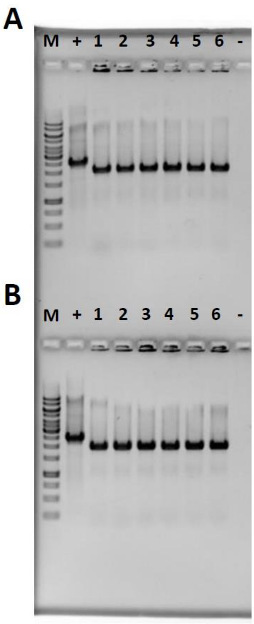

Colony PCR to check if the knockout of the AtlC worked. (A) The agarose gel with the PCR preparations of TMW 2.2515. (B) The agarose gel with the PCR preparations of TMW 2.146. (M) Marker, (+) cells without the knockout, (1-6) cells that were checked for a knockout, (-) negative control. Cells without knockout show a band at 2519 bp. Cells with the knockout show a band at 2011 bp.

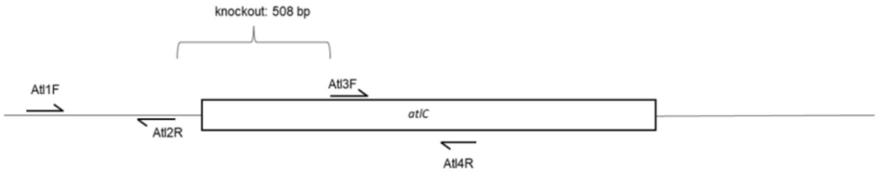

The diagram shows the atlC gene and the binding sites for the four primers used to perform the knockout. For the atlC, 508 bp were deleted.
